# Clinical and molecular epidemiology of chikungunya outbreaks during 2019–2022 in India

**DOI:** 10.1038/s41598-025-09771-9

**Published:** 2025-07-26

**Authors:** Naren Babu N, Anup Jayaram, Ujwal Shetty, Prasad Varamballi, Piya Paul Mudgal, Vikas Suri, Mini P. Singh, Kamaljeet Kamaljeet, Sachee Agrawal, Mala Kaneria, Seema Kini, Anupam Dey, Dhriti Sundar Das, Shakuntala Mahilkar, Garvita Mathur, Sakshi Chaudhary, P. Sanjai Kumar, Sharad Singh, Sweta Smita Pani, Soma Chattopadhyay, Sujatha Sunil, Baijayantimala Mishra, Jayanthi Shastri, R. K. Ratho, Anitha Jagadesh

**Affiliations:** 1 Manipal Institute of Virology, Manipal Academy of Higher Education (MAHE), Manipal, India; 2 Department of Internal Medicine, Post Graduate Institute of Medical Education and Research (PGIMER), Chandigarh, 160012 India; 3 Department of Virology, Post Graduate Institute of Medical Education and Research (PGIMER), Chandigarh, 160012 India; 4 Department of Microbiology, TNMC and BYL, Nair Charitable Hospital, Mumbai, Maharashtra 400008 India; 5 Department of Medicine, TNMC and BYL, Nair Charitable Hospital, Mumbai, Maharashtra 400008 India; 6 Department of General Medicine, All India Institute of Medical Science (AIIMS), Bhubaneswar, Odisha India; 7 Vector-Borne Diseases Group, International Centre for Genetic Engineering and Biotechnology (ICGEB), New Delhi, 110067 India; 8 Institute of Life Sciences (ILS), Nalco Square, Bhubaneswar, Odisha 751023 India; 9 Division of Neonatology, Department of Pediatrics, University of Wisconsin–Madison, Madison, Wisconsin 53706 United States of America; 10 School of Biotechnology, Kalinga Institute of Industrial Technology (KIIT) University, Bhubaneswar, Odisha 751024 India; 11 Department of Microbiology, All India Institute of Medical Sciences (AIIMS), Bhubaneshwar, Odisha 751019 India

**Keywords:** Chikungunya fever, CHIKV outbreaks, Clinical epidemiology, Dengue coinfection, Comorbidities, Spatial variation, Molecular epidemiology, Good health and well being, Diseases, Medical research

## Abstract

Chikungunya fever (CHIKF) is endemic in India, with multiple outbreaks occurring across the country since its reemergence in 2005. Suspected CHIKF patients were recruited from four clinical sites during 2019–2022, with data collected on sociodemographic, clinical, and epidemiological aspects. Sera samples were screened for IgM, IgG antibodies and viral RNA along with their neutralizing capacity. Envelope genes of Chikungunya virus (CHIKV) isolated were sequenced and further analysed. A total of 1312 suspected patients were screened during the study period; 258 patients were laboratory-confirmed with CHIKV infection. Severe clinical manifestation was observed in the patients during the viremic phase of infection. The neutralization potential was found to be increasing proportionally with the onset of illness, coinciding with the rise of IgG antibodies. Three of the four clinical sites had reported CHIKF outbreaks at different time points during the study period, and a distinct pattern of clinical presentation was observed across the sites. Phylogenetic and network analyses of E1, E2 and E3 genes from 62 CHIKV isolates demonstrated their evolution within the country. This study provides preliminary evidence of spatial and temporal variation in the clinical presentation and molecular evolution of virus in CHIKF outbreaks across India.

**Supplementary Information:**

The online version contains supplementary material available at 10.1038/s41598-025-09771-9.

## Introduction

Chikungunya fever (CHIKF) is endemic in India, with multiple outbreaks occurring across several parts of the country since its reemergence in 2005^1^. The constant reintroduction of the etiologic pathogen chikungunya virus (CHIKV) from outside the country is attributed to the circulation of new viral strains within the country, which appears to be a plausible cause of these outbreaks^2^. An important characteristic of CHIKV infection is its ability to cause outbreaks of epidemic proportions every few years, interspersed with a few years of episodic cases. With almost 480 outbreaks recorded across 30 Indian states since 2015, CHIKF has been the third most common vector-borne disease in India (IDSP, weekly outbreak reports). According to the National Centre for Vector-borne Disease Control (NCVBDC), a major outbreak of 64057 confirmed cases was recorded in 2016; since then, CHIKF has been reported in states such as Karnataka, Gujarat, Maharashtra, Madhya Pradesh, Telangana, Puducherry, Tamil Nadu, Kerala, West Bengal, Punjab, and Goa, which are among the worst hit states in the country^3^. Based on its geographical distribution, CHIKV has been classified into three major genotypes, namely, the Asian, East Central South Africa (ECSA) and West African (WA) genotypes. Notably, outbreaks in India during the 1970s involved the Asian lineage; however, after reemergence in 2005, the circulating strains were identified as being associated with the Indian Ocean (IOL) sub lineage of ECSA^1^.

Disease presentation associated with CHIKF can be divided into acute and chronic phases^4^. The acute phase is characterized by symptoms such as fever and arthralgia. Bilateral polyarthralgia is a common complication of CHIKF that typically affects small joints, such as the ankles, wrists and phalanges, as well as a few larger joints, such as the knees and elbows (WHO, 2009). Patients may develop cutaneous lesions, which frequently manifest as maculopapular rashes or oedematous or itchy skin affecting mostly the face and trunk^5^. There have also been reports of other major febrile symptoms during the acute phase, including anorexia, myalgia, nausea, vomiting, back pain, headache, diarrhea, abdominal pain, and fatigue^6^. Occasionally severe CHIKF is associated with symptoms such as ocular, renal, and neurological complications^7,8^. The disease is self-limiting and usually resolves within two weeks. However, approximately 30–40% of patients may progress to a chronic stage with persistent polyarthralgia that can persist for months to years^9^.

The current report provides a status update on the clinical and molecular epidemiology of CHIKF in India between 2019 and 2022. We provide the clinical information of CHIKF patients recruited from four clinical sites in India along with their severity, seroconversion and comorbidity conditions to understand the variation in clinical severity and chronicity of the infection across the country. We further provide information of the molecular phylogeny and evolution of the clinical CHIKV isolates from these outbreaks.

## Methods

### Patient recruitment

In this study, 1312 suspected symptomatic CHIKF patients were recruited from four clinical sites between August 2019 and December 2022 as a part of the translational research consortia (TRC) for chikungunya in India. The clinical sites included district hospitals (DHs) and tertiary care hospitals from Tamilnadu (Krishnagiri), Maharashtra (Mumbai), Punjab (Chandigarh) and Odisha (Khordha) (Supplementary Fig. 1). For the sake of convenience, the clinical sites are mentioned by the district where they are located throughout the article. All the clinical sites included were reference hospitals in the respective districts catering to all economic groups. The hospitals were selected based on factors such as the size of the hospital and surrounding catchment area, geographic representation, and operational feasibility, and the patients were recruited upon their consent following institutional ethical protocols.

Patients with clinical presentation of acute febrile illness < 15 days with arthralgia and/or arthritis and who were 12 to 75 years of age were identified as suspected CHIKF patients. Patients with known diagnoses such as malignancy, immunocompromised status, known exposure to toxins and trauma were excluded from the study. Additionally, patients who were unable to provide informed consent or assent and/or lacked a legally authorized representative available, patients who refused to provide clinical samples were also excluded from the study. Written informed consent and assent were obtained from the patient or parent/guardian (for children < 18 years old) during recruitment.

### Clinical evaluation

The patients were recruited into the study using a standard questionnaire (case report form) to retrieve basic sociodemographic, clinical, and epidemiological information (Supplementary file 2). Furthermore, disease severity was estimated using the Clinical Disease Activity Index (CDAI), and socioeconomic status was estimated by using the modified Uday Pareek scale^10^.

### Laboratory investigations

At the time of recruitment, 5 ml blood samples were collected, and serum was separated from suspected acute CHIKF cases and screened for CHIKV specific IgM antibodies using the validated CHIKjj Detect™ IgM ELISA Kit (InBios, USA) (Sensitivity − 92.4%, Specificity − 90.5%)^11^, as instructed by the manufacturer. CHIKjj Detect™ IgM ELISA Kit (InBios, USA) is known for less cross reactivity with Dengue, Zika and other flaviviruses. Additionally, CHIKV specific IgG antibody titres were estimated using indirect ELISA with purified inactivated CHIKV as the antigen, for all CHIKV positive cases as described previously^12^. The neutralizing capacity of patient sera was assessed with plaque reduction neutralization test (PRNT) in Vero cells using previously published protocols^13^. Briefly, starting with a 1:20 dilution, serum samples were serially diluted two-fold up to 10 dilutions and mixed with 50 plaque-forming units (PFUs) of lab-adapted CHIKV strain (IND/2010/DEL/01, ECSA genotype, Accession: MH124570.1) in Dulbecco’s Modified Eagle Medium (DMEM). After one hour incubation at 37﻿°C, this mixture was added to monolayer of Vero cells for 2 h at 37 °C. Cells were then overlaid with 2% carboxymethyl cellulose in DMEM containing 10% fetal bovine serum and incubated for 72 h at 37 °C with 5% CO₂. Post incubation, cells were fixed, and plaques counted. PRNT₅₀ titres were determined as the highest serum dilution that reduced plaque counts by 50%. Additionally, Dengue specific IgM antibodies using Panbio™ Dengue IgM Capture ELISA, (Abbott Diagnostics, Republic of Korea) and NS1 antigen using Panbio™ Dengue Early ELISA, (Abbott Diagnostics, Republic of Korea) were also performed for all samples.﻿

The presence of CHIKV RNA in the sera samples was further investigated by real-time reverse transcription polymerase chain reaction (RT–PCR). The amplification target was a 127-base pair (bp) fragment of the structural envelope protein E1 gene of the virus not cross reacting with other flavivirus^14^. Viral RNA was extracted from the sera samples using the Favorgen^®^ Viral RNA Extraction Kit (Favorgen, Taiwan) as instructed by the manufacturer. The primers and probe used were described earlier^14^, whereas the cycling conditions were standardized for the enzyme and buffer mix (Ambion™, Life Technologies, USA). A Ct value of < 35 was considered positive for CHIKV. All the results were analyzed using GraphPad Prism Version 6.﻿

### Sequencing of CHIKV E1, E2 and E3 genes

Structural envelope genes of CHIKV, namely, E1, E2, and E3, were amplified from positive real-time RT-PCR samples using primer sequences listed in (Table 1). Viral RNA was extracted from sera samples using the NucleoSpin RNA virus Kit (Macherey-Nagel, Germany). This RNA was then used as a template for RT-PCR with the One-Step PrimeScript RT-PCR Kit (DSS Takara Bio India Private Ltd, India). Samples showing high-intensity PCR amplicons were excised from 1% agarose gel and purified using NucleoSpin Gel and a PCR clean-up kit (Macherey-Nagel, Germany), followed by sequencing. The obtained nucleotide sequence was analyzed with published GenBank sequences using BLAST analysis on the National Centre for Biotechnology Information website (NCBI) http://www.ncbi.nlm.nih.gov/BLAST.

**Table 1:** Primer sets used in the study for CHIKV E1, E2 and E3 gene amplifications.

Gene	Product length	Genome location	Primer sequence
E1	1320	9980 to 10,003	Forward primer 5′-ATGGACGAACACGTAACAGTGATC-3′
		11,299 to 11,281	Reverse primer 5′-GTGCCTGCTGAACGACACG − 3′
E2	1271	8528 to 8552	Forward primer 5′-ATGGGCACCAAGGACAATTTCAATG − 3′
		9798 to 9777	Reverse primer 5′-GCTTTAGCTGTTCTGATGCAGC-3′
E3	533	8051to 8072	Forward primer 5′-CAGATACCCGTGCACATGAAGT-3′
		8583 to 8564	Reverse primer 5′-TGAGCTAAGTATGGTCTTGT-3′

### Phylogenetic and network analysis

The sequences were assembled by using BioEdit Sequence Alignment Editor Version 5.0.9 Program and aligned with a known CHIKV sequence representing the first CHIKV isolate (IND-KA51 strain) that re-emerged in 2006 outbreak in India (FJ000068). Additionally, CHIKV E1, E2 and E3 sequences from Bacterial and Viral Bioinformatics Resource Center (BV-BRC) that includes Indian sequences from the year 2019 to 2022 were retrieved for performing phylogenetic analysis using MEGA11 software^15^. The viral sequences obtained were aligned using ClustalW software embedded in the MEGA11 software. The phylogenetic tree was constructed using Mid-point rooted Maximum Likelihood method with 1000 bootstrap replications for evaluating the reliability of the analysis^16^. The evolutionary distances were computed using the p-distance method^17^ and are in the units of the number of base differences per site. The rate variation among sites was modelled with a gamma distribution (shape parameter = 1.21). Phylogenetic tree obtained from MEGA11 software were further edited in Figtree software for better representation. Network analysis was done by Splits Tree and Network programme using the E1, E2, E3 genes generated during the study. Snippy software pipeline were used to identify mutations in E1, E2 and E3 sequences. Each sequence was compared with reference sequence and mutations per residue positions were identified.

### Ethics statement

The study was carried out following institutional ethical approval of MAHE Manipal, PGIMER Chandigarh, TNMC Mumbai and AIIMS Bhubaneswar (Reference: MAHE EC/004/2020, PGIMER PGI/IEC/2019/000011, TNMC IEC/24/2020, and AIIMS Bhubaneswar T/EMF/Micro/19/09). All research was performed in accordance with relevant guidelines/regulations, and informed consent was obtained from all participants and/or their legal guardians. The study was performed in accordance with the Declaration of Helsinki.

## Results

### Epidemiology trends of CHIKF outbreaks in India (2019–2022)

**Fig. 1:**
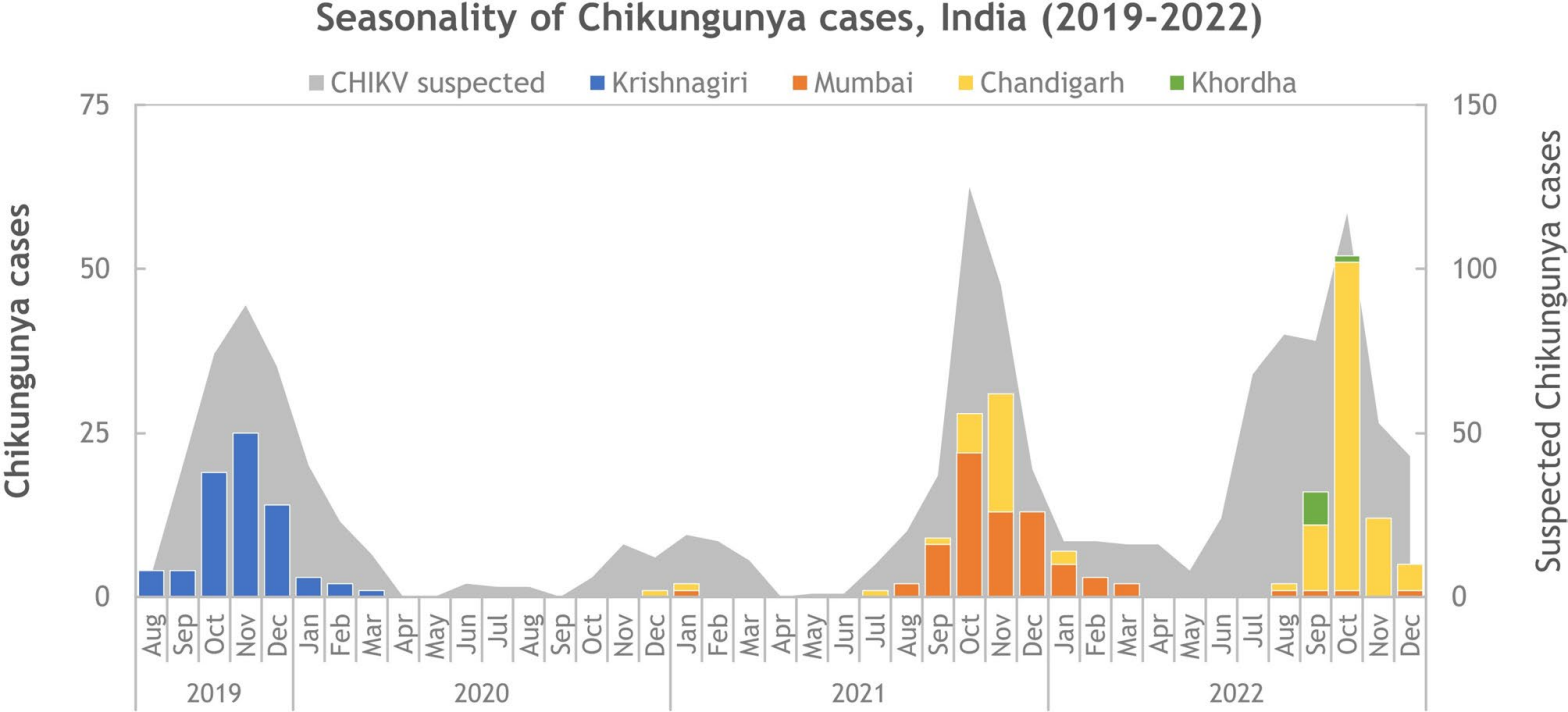
Seasonality of Chikungunya patients identified in India during the study period (Aug 2019–Dec 2022). The number of suspected cases is presented in the background as an area graph referring to the Y-axis on the right (Grey shading). The month-wise numbers of laboratory-confirmed CHIKF cases are presented as a multicolour stacked column chart referring to the Y-axis on the left (The blue-Krishnagiri district of Tamilnadu, the orange-Mumbai district of Maharashtra, the yellow-Chandigarh district of Punjab and the green-Khordha district of Odisha).

The present study screened 1312 suspected symptomatic patients with CHIKF between August 2019 and December 2022 from four clinical sites in India, namely, Krishnagiri in Tamil Nadu, Mumbai in Maharashtra, Chandigarh in Punjab and Khordha in Odisha (Supplementary Fig. 1). The screening was performed by real-time RT‒PCR and/or with IgM antibodies using the patients’ sera samples. A total of 258 patients were diagnosed with CHIKV infection (19.6%) (RT‒PCR-77, RT‒PCR + IgM-37 and IgM-144). At all the clinical sites, the CHIKF-suspected and CHIKF-confirmed cases spiked in the post-monsoon season (September-February). (Fig. 1) (Supplementary Table 1).

Of the four clinical sites, three reported CHIKF outbreaks occurred at different time periods in the years included in the study **(**Fig. 1) (Supplementary Fig. 1). In 2019, patients with 277 suspected cases were screened in Krishnigiri, of which 66 had confirmed cases. In 2021, another outbreak was reported in Mumbai after 117 patients were screened, and 59 cases were confirmed. The same year, cases were also reported in Chandigarh, albeit not in as many numbers as reported in Mumbai. However, in 2022, 158 patients were screened at the Chandigarh site, and 79 patients were confirmed (Fig. 1). However, at the Khordha site in Odisha, no CHIKF cases were recorded during these years, and a few cases (*n* = 6) were reported in 2022. Owing to the COVID-19 pandemic, all wards were converted to COVID-19 wards, and no non-COVID-19 cases were registered in 2020 (Fig. 1).

**Table 2:** Demographic characteristics of patients with CHIKF across clinical sites in India.

Demographic Characteristics		Year 2019 Krishnagiri (*n* = 66)	Year 2021 Mumbai (*n* = 59)	Year 2022 Chandigarh (*n* = 79)	Year 2022 Khordha (*n* = 6)
Age in years	Median (IQR)	30.5 (20–45)	40 (29–50)	40 (30–46)	36.5 (26–49)
	Range	12 to 65	13 to 67	13 to 70	15 to 57
Age in years (Male)	Median (IQR)	35 (21–55)	42 (27–50)	38 (31–43)	26 (15–42)
	Range	15 to 65	13 to 65	14 to 70	15 to 42
Age in years (Female)	Median (IQR)	30 (19–45)	39.5 (30–50)	41 (30–53)	49 (31–57)
	Range	11 to 65	21 to 67	13 to 67	31 to 57
	Age Group	**N (%)**	**N (%)**	**N (%)**	**N (%)**
	12 to 20	15 (22.7)	2 (3.4)	5 (6.3)	1 (16.7)
	21 to 30	12 (18.2)	14 (23.7)	14 (17.7)	1 (16.7)
	31 to 40	14 (21.2)	11 (18.6)	18 (22.8)	1 (16.7)
	41 to 50	11 (16.7)	16 (27.1)	27 (34.2)	2 (33.3)
	51 to 60	7 (10.6)	11 (18.6)	7 (8.9)	1 (16.7)
	61 to 70	7 (10.6)	5 (8.5)	7 (8.9)	0 (0)
	71 to 75	0 (0)	0 (0)	1 (1.3)	0 (0)
Gender	Male	26 (39.4)	33 (55.9)	42 (53.2)	3 (50)
	Female	40 (60.6)	26 (44.1)	37 (46.8)	3 (50)
BMI	BMI < 17.0: thinness.	8 (12.1)	1 (1.7)	0 (0)	1 (16.7)
	BMI < 18.5: underweight.	5 (7.6)	1 (1.7)	0 (0)	0 (0)
	BMI 18.5–24.9: normal weight.	42 (63.6)	30 (50.8)	7 (9)	4 (66.7)
	BMI ≥ 25.0: overweight.	8 (12.1)	14 (23.7)	5 (6.4)	1 (16.7)
	BMI ≥ 30.0: obesity	1 (1.5)	8 (13.6)	2 (2.6)	0 (0)
Socio-Economic Status	Upper	0 (0)	0 (0)	0 (0)	0 (0)
	Upper Middle	0 (0)	0 (0)	0 (0)	0 (0)
	Middle	2 (3)	27 (45.8)	55 (70.5)	3 (50)
	Lower Middle	61 (92.4)	29 (49.2)	17 (21.8)	3 (50)
	Lower	3 (4.5)	3 (5.1)	6 (7.7)	0 (0)

**Fig. 2:**
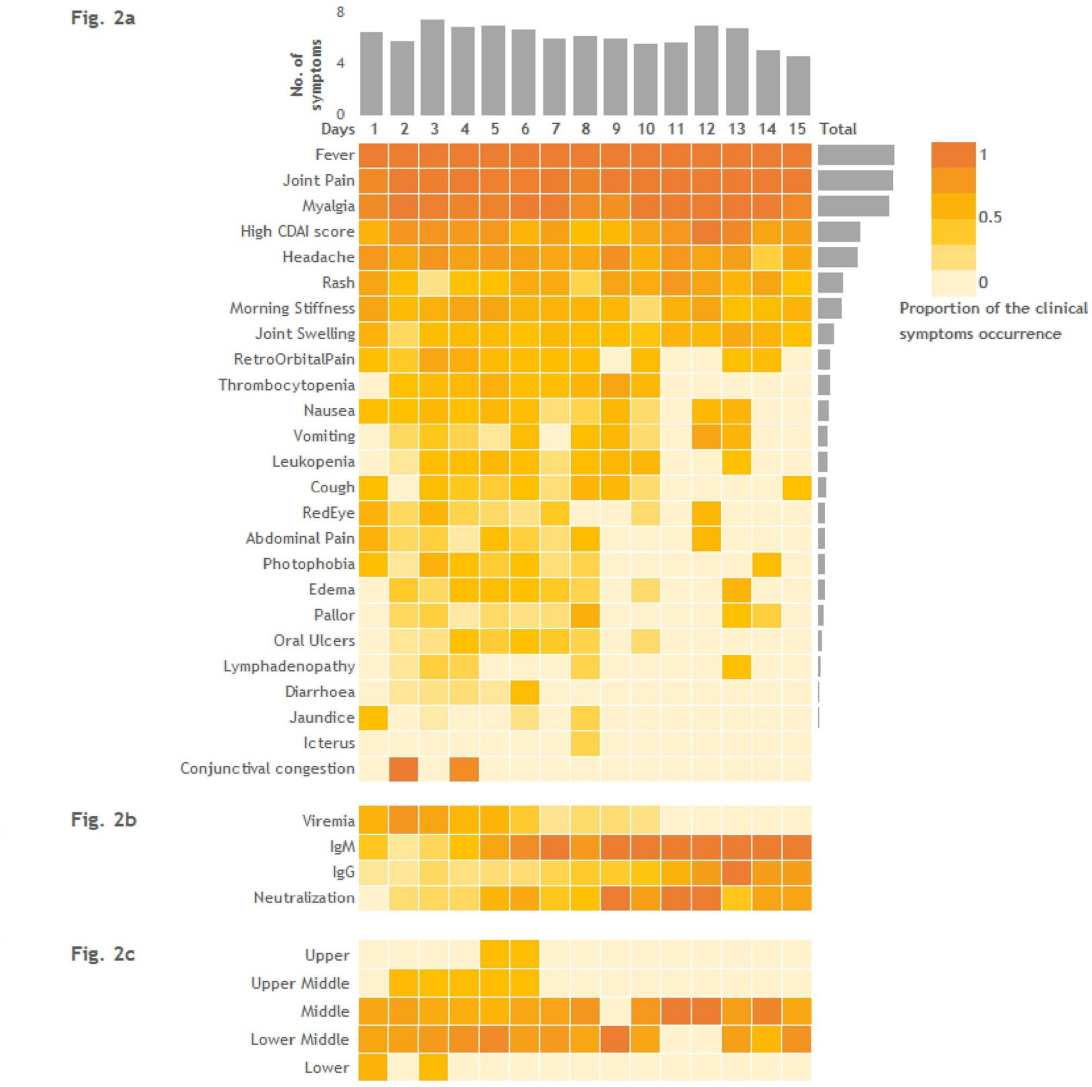
Clinical symptoms of chikungunya were recorded at the time of enrolment. (**a**) The proportions (0 to 1) of patients who presented with clinical symptoms are presented in a heatmap. The scale at the right top corner represents the proportion of patients with symptoms occurring in the increasing shade of orange (mildest shade: 0 and darkest shade: 1). (**b**) The proportions (0 to 1) of patients identified as reactive to the laboratory assays are presented in a heatmap. The scale at the right top corner represents the proportion of patients with symptoms occurring in the increasing shade of orange (mildest shade: 0 and darkest shade: 1). (**c**) The proportions (0 to 1) of patients identified with CHIKF categorically based on their socio-economic status are presented in a heatmap. The scale at the right top corner represents the proportion of patients with symptoms occurring in the increasing shade of orange (mildest shade: 0 and darkest shade: 1).

As per the case definition, patients were recruited between 1 and 15 days after the onset of illness. More than 80% of the patients visited the clinical site within 8 days of the onset of illness, with an estimated average of 5 days. Furthermore, a greater number of clinical symptoms were observed between 3 and 6 days after onset of illness, after which the symptoms progressively decreased thereafter. Intriguingly, an upwards shift in the number of blips was observed on the 12th or 13th day (Fig. 2a).

The sociodemographic data of the CHIKF patients were analysed, and > 95% of the CHIKF patients were classified as middle or lower-middle socioeconomic status (Table 2). At the Krishnagiri site, > 90% of the patients had a lower-middle socioeconomic status. At the Mumbai and Khordha sites, 50% of the patients were middle-level and 50% were lower-middle-level socioeconomic status. In contrast, > 70% of the patients from Chandigarh had a middle socioeconomic status. Interestingly, the number of patients admitted to the hospital within 6 days of illness was the highest (100%) among the upper socioeconomic status groups, which was further reduced by 50.5% in the patients with a lower middle socioeconomic status (Fig. 2c).

### Clinical profile of CHIKF patients across the study sites

Upon analysing the clinical presentations of the CHIKF patients, it was observed that fever, joint pain and myalgia were the common clinical symptoms across the clinical sites (Table 3; Fig. 2a). However, atypical symptoms such as headache and morning stiffness were prevalent in Krishnagiri and Mumbai, and retro-orbital pain was observed mostly in Krishnagiri (Table 3; Fig. 2a). Interestingly, rashes were not reported at most of the clinical sites, except for Chandigarh (43%) (Table 3), where ~ 75% of the rashes appeared as macular or maculopapular lesions^18^.

At the time of enrolment, more than 60% of the patients were categorized into the high disease activity (severe) group according to the CDAI classification (Table 1); however, Mumbai recorded severe cases as high as 70%. The severity of the disease was greatest in the patients who visited the hospital during the viremic phase of infection. Importantly, the patients categorized as severe exhibited a broad spectrum of clinical symptoms compared to the non-severe cases. (Fig. 2a).

**Table 3:** Comparison of clinical characteristics between CHIKF outbreaks in India.

Clinical parameters	Year 2019 krishnagiri (*n* = 66) *N* (%)	Year 2021 Mumbai (*n* = 59) *N* (%)	Year 2022 Chandigarh (*n* = 79) *N* (%)	Year 2022 Khordha (*n* = 06) *N* (%)
Fever	66/66 (100)	59/59 (100)	79/79 (100)	6/6 (100)
Joint pain	66/66 (100)	59/59 (100)	79/79 (100)	6/6 (100)
Myalgia	64/66 (97)	56/59 (94.9)	78/79 (98.7)	6/6 (100)
High CDAI Score	45/66 (68.2)	42/59 (71.2)	45/79 (57.7)	3/6 (50)
Moderate CDAI Score	7/66 (0.1)	17/59 (28.8)	34/79 (43)	2/6 (33.3)
Low CDAI Score	12/66 (0.2)	0/59 (0)	0/79 (0)	1/6 (16.7)
Headache	63/66 (95.5)	32/59 (54.2)	9/79 (11.4)	6/6 (100)
Morning stiffness	44/66 (66.7)	22/59 (37.3)	1/79 (1.3)	3/6 (50)
Retro orbital pain	42/66 (63.6)	2/59 (3.4)	3/79 (3.8)	2/6 (33.3)
Photophobia	23/66 (34.8)	3/59 (5.1)	0/79 (0)	0/6 (0)
Nausea	21/66 (31.8)	7/59 (11.9)	5/79 (6.3)	0/6 (0)
Cough	18/66 (27.3)	6/59 (10.2)	0/79 (0)	0/6 (0)
Vomiting	16/66 (24.2)	2/59 (3.4)	3/79 (3.8)	0/6 (0)
Joint swelling	15/66 (22.7)	7/59 (11.9)	16/79 (20.3)	1/6 (16.7)
Red eye	14/66 (21.2)	6/59 (10.2)	1/79 (1.3)	1/6 (16.7)
Pallor	14/66 (21.2)	0/59 (0)	0/79 (0)	0/6 (0)
Edema	11/66 (16.7)	2/59 (3.4)	12/79 (15.2)	0/6 (0)
Icterus	10/66 (15.2)	0/59 (0)	0/79 (0)	0/6 (0)
Lymphadenopathy	10/66 (15.2)	0/59 (0)	0/79 (0)	0/6 (0)
Oral ulcers	10/66 (15.2)	0/59 (0)	7/79 (8.9)	0/6 (0)
Abdominal pain	9/66 (13.6)	5/59 (8.5)	1/79 (1.3)	0/6 (0)
Diarrhoea	6/66 (9.1)	0/59 (0)	2/79 (2.5)	0/6 (0)
Rash	1/66 (1.5)	6/59 (10.2)	34/79 (43)	0/6 (0)
Jaundice	0/66 (0)	1/59 (1.7)	0/79 (0)	0/6 (0)

More than 60% of CHIKF patients experiencing joint pain reported discomfort in both small and large joints. The specific joints affected varied among patients; notably, a large majority of those with severe symptoms (88%) reported pain in both types of joints. Joint pain involving both small and large joints was found to be more prevalent as patients aged, ranging from 33% in the youngest to 76% in the oldest age-group, with no significant gender difference observed. Arthralgia of large joints alone was more common (67%) in the severe group than in the non-severe group.

### Seroconversion and Seroprevalence of CHIKV across the study sites

The majority (50–80%) of the CHIKF patients who visited clinical sites between 1 and 6 days had viraemia in their sera samples. Moreover, the majority of patients exhibited detectable IgM antibody levels beginning on day 3, with most seroconverting by day 6 (Fig. 2b). Approximately 70% of the patients developed IgM antibodies at the time of recruitment, and those patients presented with a higher rate of rashes and joint swelling (Fig. 2a and b). All the 258 sera sample were also screened for IgG antibodies. The study identified IgG antibodies in approximately 40% of CHIKF patients who visited clinical sites during the post viraemic phase, i.e., > day 10 post onset of illness (Fig. 2b). IgG antibody levels began to rise around day 5 following the onset of illness, with a marked increase observed from day 7 onward. Furthermore, the neutralization capacity of the patients’ sera was assessed using Indian CHIKV strain (IND/2010/DEL/01, ECSA genotype, Accession: MH124570.1) and a threshold of PRNT_50_ titer of ≥ 1:20 considered indicative of seropositivity. The neutralization potentials of the patients’ sera began to increase shortly after the rise in IgG levels, typically between days 6 and 7, and reached high titers from day 9 onward, suggest a strong correlation between IgG and the neutralization capacity of the patients’ sera (Fig. 2b).

### Coinfections and comorbidities among the CHIKF patients

**Fig. 3:**
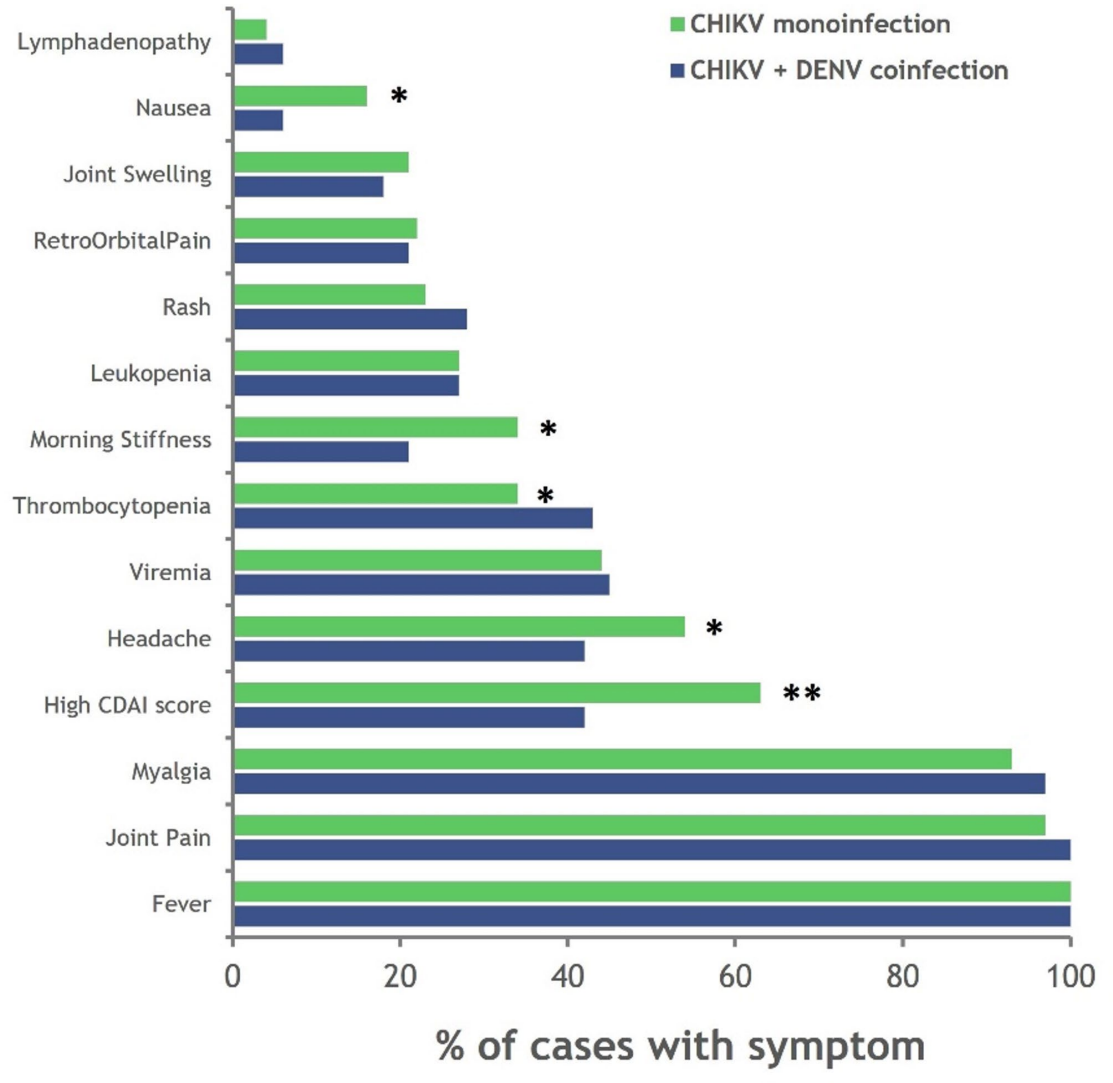
Clinical symptoms of patients with dengue coinfection. The line graph represents the differences in the percentage (0 to 100) of clinical symptoms between CHIKV mono-infections (Green) and coinfections with DENV (Blue). The statistical significance was estimated using Fisher’s exact test (p-value presented in the graphs: <0.05-* and < 0.01-**).

The study also revealed that 33 of the 258 (12.8%) CHIKF patients had coinfection with dengue virus (DENV). Like in CHIKF patients, coinfections occurred equally in both genders and predominantly affected individuals in the lower-middle socioeconomic category. Nearly 45% of the coinfected patients were identified with the dengue NS1 antigen, whereas the rest were identified via the presence of IgM antibodies. The study recruited 10 patients with CHIKV viremia and DENV antigen, indicating active infections of both viruses, i.e., those positive for CHIKV via real-time RT‒PCR and for the Dengue NS1 antigen. The frequency of thrombocytopenia, rash, myalgia, and lymphadenopathy was greater in the dengue coinfection patients than in the CHIKV monoinfection patients, whereas severe arthralgia (CDAI score > 22) was observed at a greater rate along with headache, morning stiffness, joint swelling, and nausea in case of CHIKV monoinfection (Fig. 3). Typical dengue symptoms such as thrombocytopenia were more frequently observed in co-infected patients, whereas severe arthralgia was more commonly associated with CHIKV mono-infection.

**Fig. 4:**
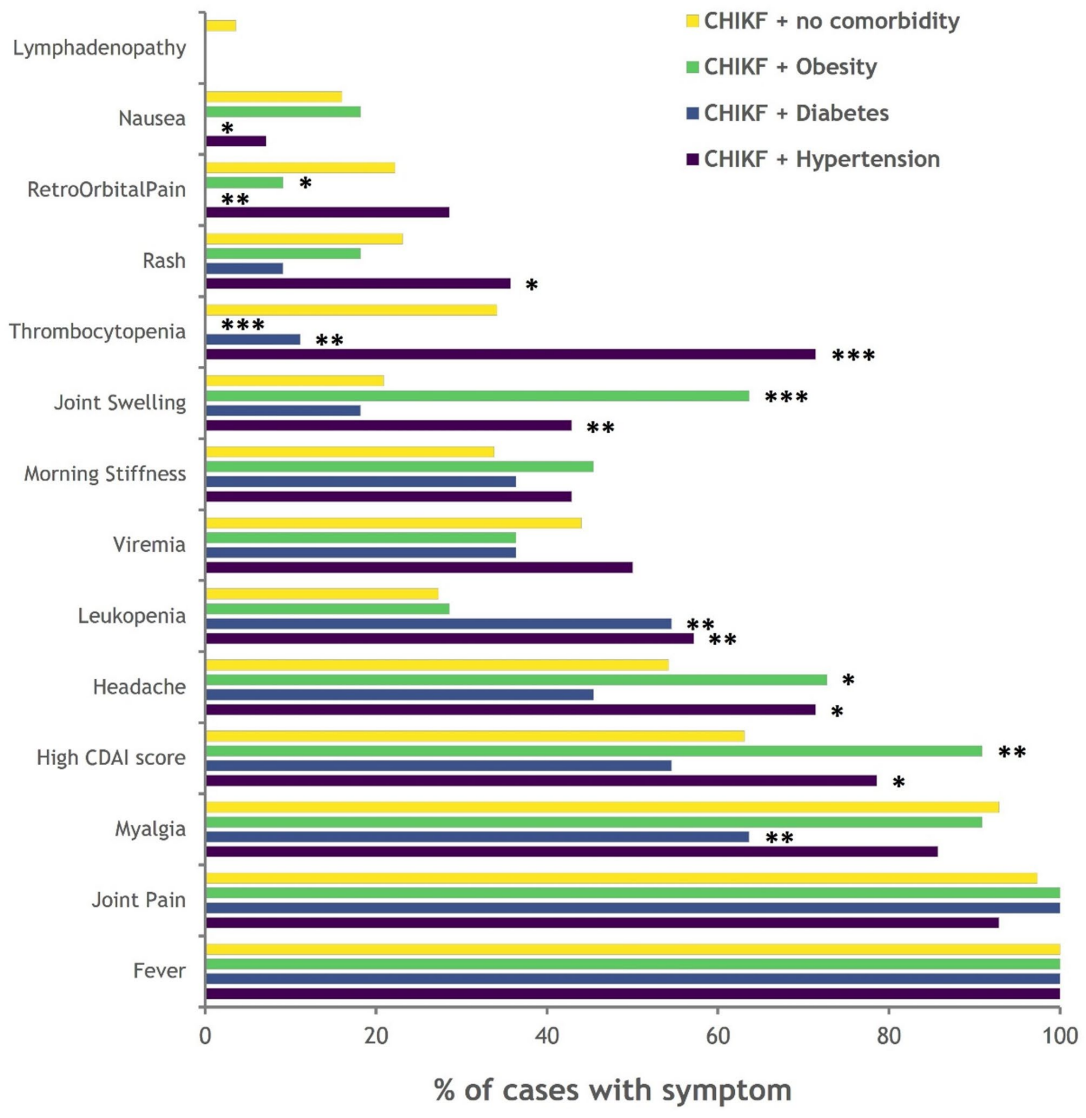
Clinical symptoms of CHIKF patients with comorbidity. The line graph represents the differences in the percentage (0 to 100) of clinical symptoms between CHIKF + no comorbidity (Yellow), CHIKF + Obesity (Green), CHIKF + diabetes (Blue), and CHIKF + Hypertension (Purple). The statistical significance was estimated using Fisher’s exact test (p-value presented in the graph: <0.05-*, < 0.01-** and < 0.001-∗∗∗).

Among the CHIKF patients, 14.3% (37/258) had at least one comorbid condition, such as hypertension (*n* = 14), diabetes (*n* = 11), obesity (*n* = 11), asthma (*n* = 3), myocarditis (*n* = 3) or liver cirrhosis (*n* = 1). The CHIKF patients with comorbidities (~ 60%) suffered from severe arthralgia (Fig. 4). Patients with hypertension or obesity increasingly experienced severe arthralgia, joint swelling, and headache (Fig. 4). It was also observed that 91% of the obese patients presented severe arthralgia (Fig. 4). In contrast, the underweight individuals had experienced other symptoms, such as headache, retro-orbital pain, photophobia, vomiting, abdominal pain and diarrhea more frequently. Among hypertensive patients, during CHIKF, haematological parameters such as WBC and platelet counts are decreased, whereas the symptoms including rashes, leukopenia, and thrombocytopenia, are increased (Fig. 4).

### Phylogenetic analysis of envelope genes of CHIKV isolates from the outbreaks

**Fig. 5:**
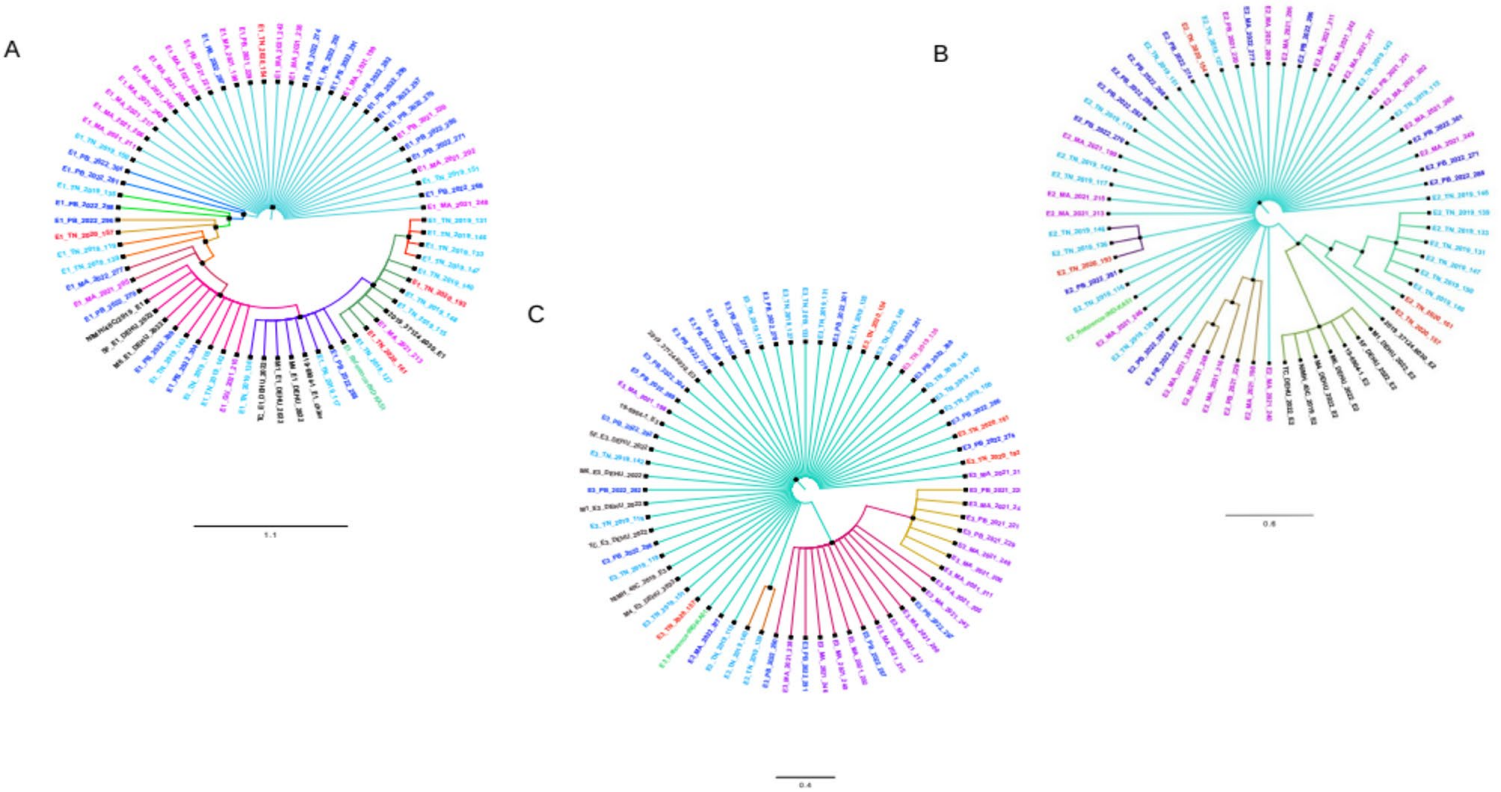
Mid-point rooted Maximum likelihood phylogenetic tree for E1, E2 and E3 sequences. Lab generated datasets has been highlighted in different colours on collection year basis. Sequence from year 2019 are highlighted in sky blue colour. Sequences which were collected in 2020 are highlighted in red colour. Sequences from year 2021 are highlighted in magenta colour while reference genome from 2006 (FJ000068), the very first sequence which were submitted to the database after re-emergence of chikungunya in India is highlighted in green colour. 2022 sequences are highlighted in blue colour. Sequences which are collected from database has been remained in black colour. Taxon labels include gene name, collection state, collection year and virus strain (pool number). The scale bar indicates percent divergence between sequences (**A**) Phylogenetic tree showing the clustering of E1 sequences. (**B**) Phylogenetic tree showing the evolutionary relationship between E2 sequences. (**C**) Evolutionary relatedness between E3 sequences from 2019–2022.

Among the 144 RT-PCR confirmed CHIKF cases from the outbreaks, 62 samples that were representative of the geographical locations through 2019 to 2022 were sequenced and the sequences were subjected to phylogenetic and mutation analyses. The CHIKV E1, E2 and E3 envelope genes were sequenced and the sequences post curation, along with eight additional sequences retrieved from public database, were subjected to phylogenetic analysis. The analysis revealed all Indian CHIKV isolates belonged to ECSA genotype. Further, gene-wise analysis revealed the following clustering. The CHIKV E1 gene sequences (*n* = 61) clustered into two distinct clusters, one of which further formed several smaller sub clusters. The Maharashtra 2021 and Punjab 2022 outbreak samples primarily clustered together whereas the samples from Tamil Nadu 2020 spread across the clusters indicating variations amongst these sequences. Similarly, distinct clusters were observed amongst the Tamil Nadu 2019 outbreak samples with a few sequences distributed amongst other small clusters (Fig. 5a). The E2 gene sequences (*n* = 56) formed four distinct clusters with most of the Tamil Nadu 2019 samples clustering together. Samples from the Maharashtra 2021 and Punjab 2022 outbreaks exhibited more variations in this gene and grouped in multiple clusters (Fig. 5b). It is interesting to note that the Maharashtra 2021 outbreak samples were quite distinct those sequences obtained during a 2022 outbreak from the public dataset from the same state (Fig. 5b sequences in black fonts). The E3 envelope genes showed the minimum variations amongst the samples analysed (*n* = 60) with most of the samples clustering together, and the Maharashtra 2021 samples forming two smaller clusters along with a few Punjab 2022 samples in one of the subclusters (Fig. 5c). In addition, we performed a gene-wise mutational analysis on all laboratory-generated CHIKV E1, E2 and E3 sequences to determine amino acid conservation. Mutations were found to be equally distributed across all three genes with a slight preference towards the E1 gene. E1 sequences showed 67 mutations; the highest frequency mutations observed were V84F, Y93N, C94G, E99K, Q102H, K110N, E117K, A121P, R196K, I203F and V213F (Supplementary file 3). These mutations covered maximum sample collection sites. 50 mutations found in E2 sequences; K66Q, A76T. P133L, K252R mutations were in the highest frequencies (Supplementary file 3). E3 sequences had 7 mutations, of which mutations E28K, V42I, P59S were three major mutations that were found to be present in maximum (Supplementary file 3).

These findings provide valuable insights into the evolutionary dynamics and genetic diversity of CHIKV strains circulating in India, which have been associated with severe symptoms such as fever and joint pain. The partial gene nucleotide sequences of the CHIKV isolate from patients 99–100% nucleotide identity to one another and showed paired identity at the nucleotide level with all the Indian strains ranging from 98.3 to 99.9%. Furthermore, these CHIKV partial E1 gene sequences also shared greater than 99% nucleotide homology with sequences of CHIKV isolates available in database from 2019 to 2022, within this ECSA lineage.

### Network analysis of envelope genes of CHIKV clinical isolates from the outbreaks

**Fig. 6:**
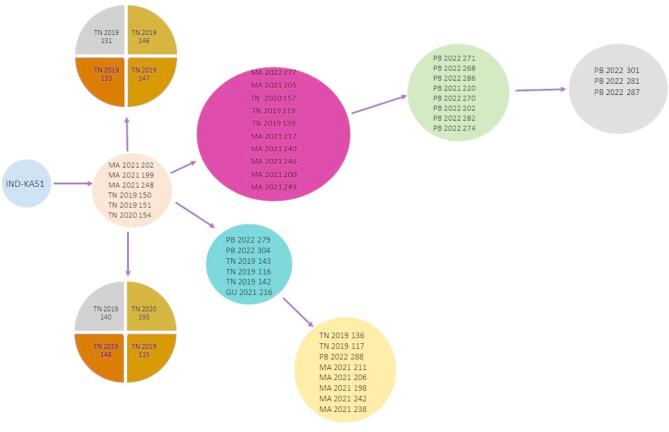
Genetic network analysis of E gene region of Indian CHIKV isolates. Nomenclature includes state name, collection year and lab generated virus strain name. Networks are labelled by years of sample collection (2019–2022) and by geographic origin (Maharashtra, Tamil Nadu, Punjab, Gujrat). The length of network connections reflects the number of mutations.

The CHIKV envelope genes, E1, E2 and E3 were further subjected to median joining network analysis to establish the genetic connections amongst the circulating strains between 2019 and 2022 based on the mutations present in the sequences (Fig. 6). The analysis revealed that the samples were distributed into eight nodes evolving from the ancestral strain of 2006. The samples from the Tamil Nadu 2019 outbreak were found to be distributed in six distinct nodes along with the samples of the other outbreaks showcasing the evolving nature of the isolates from this region. Whereas, the outbreaks for Maharashtra 2021 and Punjab 2022 appeared to be more clonal in their origin and exhibited similar mutations across the samples. Furthermore, the samples from the regions over the years appeared to have similar mutations indicating that a clonal expansion of strains of the preceding year might have led to the outbreaks in the following years.

## Discussion

The study examined the pattern of CHIKF outbreaks across India from 2019 to 2022, revealing significant spatial and temporal variations. Among febrile cases with typical joint pain screened in different parts of India as part of the study, 19.6% were diagnosed with CHIKV infection, which is approximately 4.7 to 14.2% higher than the national prevalence recorded during the time-period^1,3^. Cases tend to peak during the post-monsoon season (September-February), a trend noted in other CHIKF-prone countries^19^, attributed by increased breeding of vector mosquitoes namely, *Aedes species*. The average age of CHIKF patients ranged between 30 and 40 years, with an equal gender distribution across all clinical sites, consistent with previous reports indicating CHIKF affects individuals of all ages and genders, particularly those in their productive working years^20^. Further, the sociodemographic status of CHIKF patients, revealed that over 95% of them belonged to the middle or lower-middle socioeconomic status, aligning with findings reported previously in India^21^. The CHIKF patients in the present study showed a gradual emergence of IgG antibodies in their sera samples against the virus, typically starting from the day-10 after the onset of illness, reflecting a usual pattern of seroconversion^20,22^.

These findings are consistent with previous studies demonstrating that IgG antibodies play a key role in mediating virus neutralization during CHIKV infection^23^. Our earlier investigations further support this association, indicating that neutralizing IgG antibodies are present within the first two weeks of illness and become more potent during the convalescent phase, typically around one month post-infection^13^. Together, these findings highlight the pivotal role of IgG antibodies in the humoral immune response against CHIKV and support their potential as correlates of protective immunity.

An assessment of disease severity among individuals affected during the outbreaks revealed notable patterns across different regions. Over 60% of individuals with CHIKF experienced severe disease, though the proportion varied across geographic regions. The severity of the illness peaked among patients who sought hospital care during the viraemic phase of infection (Day 1–6), a pattern also observed in a study by Imad et al. during the 2019 outbreak in Thailand^24^. Though underlying comorbidities such as obesity, hypertension and diabetes were observed to exacerbate the severity in CHIKF patients^25–27^, a varied combination of symptoms found prevailing across the geography through the time. Variations in clinical presentation, including an increase in atypical symptoms like headache, morning stiffness and retro-orbital pain and a decrease in typical symptoms like rashes, may be attributed to the presence of different strains of CHIKV circulating across various clinical sites and warrants further in-depth studies.

Individuals with Dengue coinfections exhibit typical symptoms of both dengue and CHIKF^19,28^. Coinfection patients experienced a higher occurrence of thrombocytopenia, rash, myalgia, and lymphadenopathy compared to those with CHIKV mono-infection, while severe joint pain was more prevalent in mono-infection cases^19^.

The partial envelope genes sequence analysis revealed that CHIKV isolates belong to ECSA genotype, the prevailing genotype present in India since its resurgence in 2005^29^, which over time has evolved, leading to multiple outbreaks of CHIKV^29,30^. The 2019–2021–2022 outbreak isolates of the present study formed unique phylogenetic clusters providing clues to the evolution of the virus across the country over the years. Furthermore, network analysis of the sequence revealed that the Tamil Nadu 2019 outbreak viral strains are diverse and evolving, while the Maharashtra 2021 and Punjab 2022 strains showed clonal amplification of strains circulating in the region. The display of a varied spectrum of clinical symptoms in the patients of the different outbreaks coinciding with distinct genetic signatures provide functional attributes of these mutations in CHIKV pathogenesis. Network analysis of the sequences highlighted unique amino acid substitutions in the E1, E2, and E3 envelope glycoproteins that might have a functional significance and warrants further in-depth focussed studies. Previous studies have demonstrated a correlation between viral genetic mutations and alterations in neutralization sensitivity of CHIKV, suggesting that specific changes in viral envelope proteins can modulate immune recognition and potentially influence disease severity^31,32^. Among these, the E2 glycoprotein is the most variable and immunodominant of the CHIKV envelope proteins and is a principal target of both IgM and IgG antibodies. Neutralizing antibodies predominantly bind to domains A and B domain of E2, with domain B-directed antibodies being broadly neutralizing across different CHIKV genotypes^23,33,34^. Given the critical role of these structural proteins in viral entry and immune recognition, evaluating the functional relevance of these mutations in the context of neutralization capacity may provide insights into their potential impact on immune escape and pathogenesis.

Our findings suggest positive selection of the mutations owing to prolonged presence of these strains in the region. Our finding suggests sufficient evidence for mutational changes in E1, E2 and E3 genes of CHIKV and their role in divergent evolution of CHIKV. We identified novel mutations on E1, E2 and E3 genes in the circulating strain of CHIKV. While mutation on the E2 252 position have been previously reported from an outbreak in Pakistan i.e. K252Q^35^ while in our study, at 252 position we observed K252R at the same site. Double mutations in CHIKV E1 (A226V) and E2 (K252Q) proteins have been linked to improved fitness in *A. albopictus* mosquitoes and have been discovered to be involved in virus adaptation in local mosquito species^36^.

Though targeted sequencing of the clinical isolates have provided some insights regarding the mutation status of the envelop genes, whole genome analysis is imperative to understand the functional relevance of these mutations and to better understand the evolution of CHIKV from the different outbreak sites. These findings further confirm the presence of different clinical manifestations in different outbreak and geographical regions. Previous studies have also shown that genetic evolutions change the patterns of clinical manifestations leading to severe consequences of CHIKF with epidemic potential^30,37–40^. Previous studies involving exhaustive sequence analyses have determined that genetic evolution of circulating CHIKV is responsible for outbreaks in Indian states^2^.

Overall, the data showed spatiotemporal variability in CHIKF outbreaks at all clinical sites during the study period. The re-emergence of CHIKV to certain regions has been linked to similar variations, which are primarily caused by a combination of genetic variation, population immunity, and vector propagation^41,42^. While the research provides preliminary evidence linking the diverse symptom presentations, variability in severity and neutralization capacities to genetic heterogeneity, further investigations on the whole genome using next generation sequencing is vital. This advanced approach will help elucidate the role of genetic mutations in the observed clinical variability, thereby enhancing our understanding and management of Chikungunya and related arboviral diseases.

## Conclusion

This clinical and epidemiological study on Chikungunya in India have revealed significant variations among the outbreaks across different times and locations. The findings indicate a higher incidence of CHIKF among Indian population than previously estimated. The study also highlights dengue coinfections among the CHIKF cases, this underscores the need for intensified febrile illness surveillance for arboviral diseases, particularly during post-monsoon seasons. The study identified a diverse symptom combinations and severities throughout the country emphasising the clinical diagnosis of the CHIKF should encompass a broader spectrum of symptoms.

## Electronic supplementary material

Below is the link to the electronic supplementary material.

Supplementary Material 1

Supplementary Material 2

Supplementary Material 3

Supplementary Material 4

## Data Availability

The datasets used and/or analysed during the current study are available from the authors upon reasonable request and with permission of Institutional IEC. The request should be sent to Dr Anitha J (anitha.j@manipal.edu) as the manuscript’s corresponding author.
