# Supplementary material for: Clinical and molecular epidemiology of chikungunya outbreaks during 2019–2022 in India
Source: Sci Rep. 2025 Jul 26;15:27280. doi: 10.1038/s41598-025-09771-9 (PMC12297696; doi:10.1038/s41598-025-09771-9)

**Supplementary Files**

**Supplementary Table 1: Year-wise chikungunya positivity among clinical sites in India (2019–2022)**

|  | **Krishnagiri** | | | **Mumbai** | | | **Chandigarh** | | | **Khordha** | | |
| --- | --- | --- | --- | --- | --- | --- | --- | --- | --- | --- | --- | --- |
|  | **IgM** | **IgM & PCR** | **PCR** | **IgM** | **IgM & PCR** | **PCR** | **IgM** | **IgM & PCR** | **PCR** | **IgM** | **IgM & PCR** | **PCR** |
| **2019** | **22** | **12** | **32** | **0** | **0** | **0** | **0** | **0** | **0** | **0** | **0** | **0** |
| **2020** | **0** | **1** | **5** | **0** | **0** | **0** | **1** | **0** | **0** | **0** | **0** | **0** |
| **2021** | **0** | **0** | **0** | **42** | **8** | **9** | **14** | **11** | **2** | **0** | **0** | **0** |
| **2022** | **0** | **0** | **0** | **12** | **0** | **2** | **47** | **5** | **27** | **6** | **0** | **0** |

**Supplementary Figure 1: Chikungunya Cases in India by Clinical Site: Year-wise Distribution on Dot Density Map**


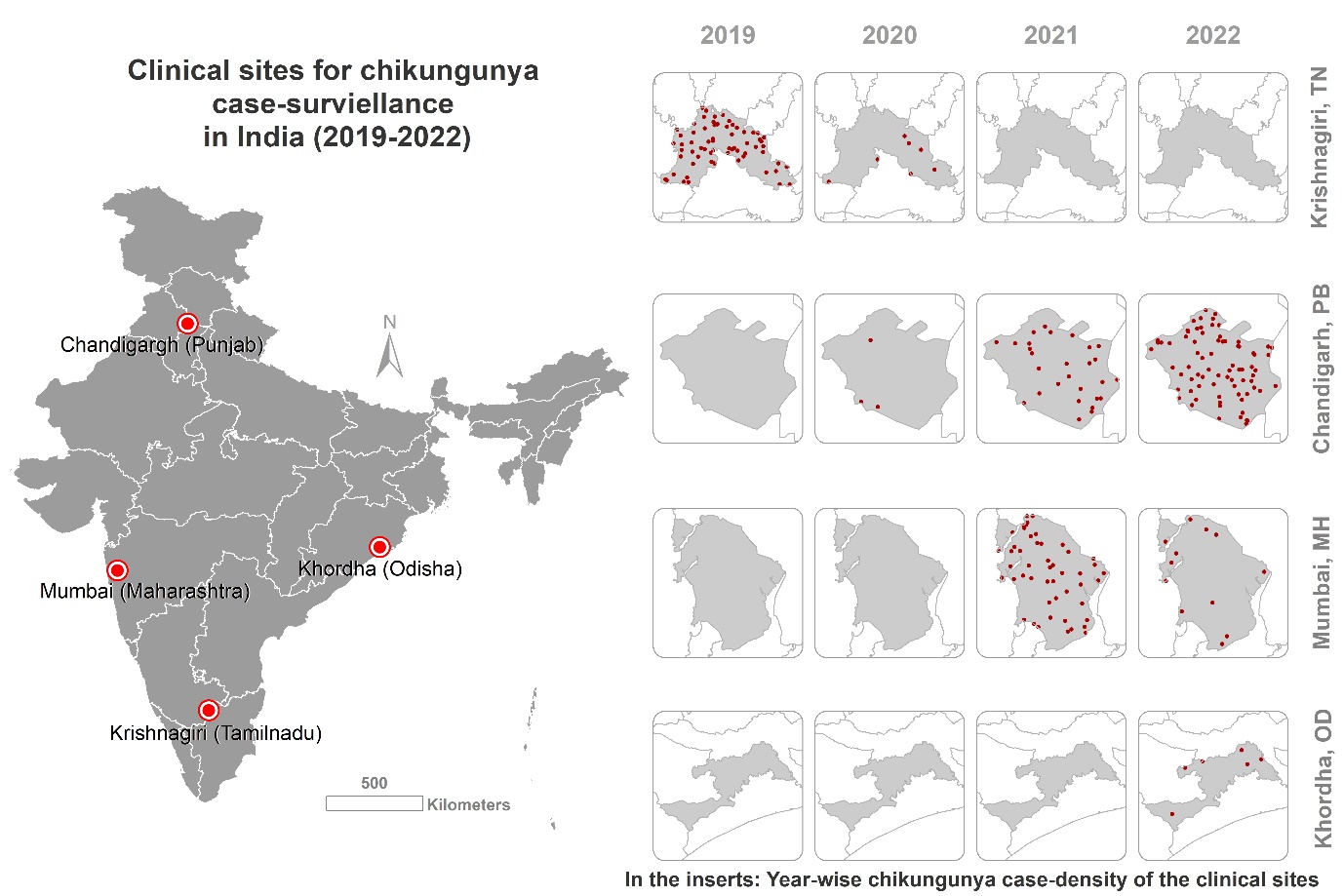

Supplement: Supplementary file 2 — Supplementary Material 2 [file 41598_2025_9771_MOESM2_ESM.docx]
