# Supplementary material for: Clinical and molecular epidemiology of chikungunya outbreaks during 2019–2022 in India
Source: Sci Rep. 2025 Jul 26;15:27280. doi: 10.1038/s41598-025-09771-9 (PMC12297696; doi:10.1038/s41598-025-09771-9)
Supplement: Supplementary file 3 — Supplementary Material 3 [file 41598_2025_9771_MOESM3_ESM.pdf]

QUESTIONNAIRE

Assign Barcode/Study ID ____

BASIC QUESTIONS

1. Name* ____
2. Age in years* ____
3. Gender* ____
4. State* ____
5. District* ____
6. Village ____
7. Ward ____
8. Pin Code* ____
9. Landmark ____
10. Primary Phone No*.: ____
11. Secondary Phone No.: ____

CRF QUESTIONS

12. Aadhar No.: ____________________
13. Date of Birth: ____________________
14. Marital Status: ____________________
15. If Married Female, 14a. Currently pregnant If Yes, 14b. Weeks __
16. Major Occupation (select from list) : ________________________
    16a. Secondary occupation : ________________________

List of occupations:

Unemployed	Skilled labourer	Manual labourer (Agriculture)
Housewife	Unskilled labourer	Manual labourer (Masonry)
Office worker	Other professionals	Shepard
Student	Farmer	Meat Handler
Health care worker		
Teacher		
Other (Specify)		

TRANSLATIONAL RESEARCH CONSORTIUM FOR CHIKUNGUNYA VIRUS

SOCIO ECONOMIC STATUS (UDAY PAREEK REVISED SCALE 2017)

**Caste**	
Schedule caste	1
Lower caste	2
Artisan caste	3
Agriculture caste	4
Prestige caste	5
Dominant caste	6

**Occupation**	
None	0
Labourer	1
Caste Occupation	2
Business	3
Independent profession	4
Cultivation	5
Service	6

**Education**	
Illiterate	0
Can read only	1
Can read and write	2
Primary	3
Middle	4
High School	5
Graduate	6
And above	7

**Land**	
No land	0
Less than 1 acre	1
1-5 acre	2
5-10 acre	3
10-15 acre	4
15-20 acre	5
20 and above	6

**Social Participation**	
None	0
Member of one organization	1
Member of more than one organization	2
Office holder in such an organization	3
Wide public leader	4

**House**	
No house	0
Hut	1
Kutcha house	2
Mixed house	3
Pucca house	4
Mansion	5

**Farm Power**	
No draught animals	1
1-2 draught animals	2
3-4 draught animals	4
5-6 draught animals	6

**Material possessions**	
Bullock cart	0
Cycle	1
Radio	2
Chairs	3
Mobile Phone	4
Television	5
Refrigerators	6

**Family Type**	
Single	1
Joint	2
Extended	3
Size up to 5	2
Any other distinctive features	2

**Grade**	**Category**		**Scale**
A	Upper class		Above 43
B	Upper middle class		33-42
C	Middle class		24-32
D	Lower middle class		13-23
E	Lower class		Below 13

CLINICAL HISTORY

17. Date of Admission: __________________
18. Date of Sample Collection/Recruitment: __________________
19. Date of Onset of Illness (DOI): __________________
20. What was the duration of fever as on today (the date of recruitment)? _____ days
21. Did you have headache within few days before/after onset of fever? (Headache) **Y | N**
22. Did you notice any colour change in your skin - a rash within few days before/after onset of fever? (Rash) **Y | N**   If Yes, 22a. Site _______________________
23. Did you have cough within few days before/after onset of fever? (Cough) **Y | N**
24. Are you troubled by bright light? (Photophobia) **Y | N**
25. Did you have pain behind your eyeball especially while moving eyes? (Retro orbital pain) **Y | N**
26. Had your eye(s) become red and irritable within few days before/after onset of fever? (Red eye) **Y | N**
27. Did you experience generalized body ache within few days before/after onset of fever? (Myalgia) **Y | N**
28. Did you experience Pain in your joints within few days before/after onset of fever? (Joint pain) **Y | N**

If Yes, 27a. Location : ☐ Small Joints ☐ Large Joints ☐ Both

27b. **CDAI SCORE**

**JOINT**	**LEFT**		**RIGHT**	
	**SWOLLEN**	**TENDER**	**SWOLLEN**	**TENDER**
**SHOULDER**				
**ELBOW**				
**WRIST**				
**MCP 1**				
**MCP 2**				
**MCP 3**				
**MCP 4**				
**MCP 5**				
**PIP 1**				
**PIP 2**				
**PIP 3**				
**PIP 4**				
**PIP 5**				
**KNEE**				
**TOTAL**	**TENDER:**		**SWOLLEN:**	

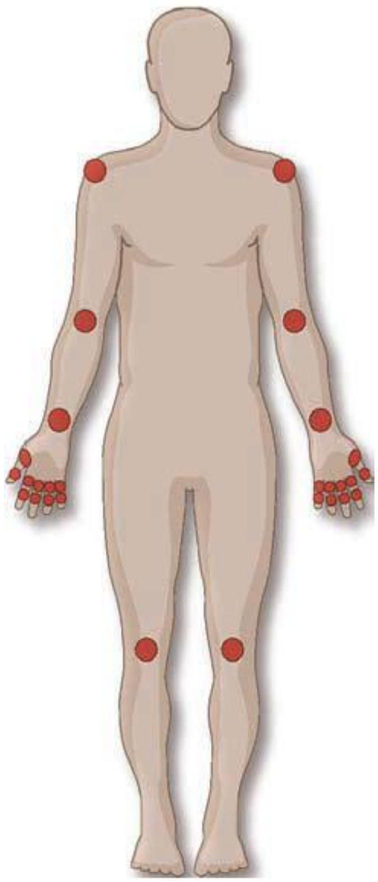

Patient Global Assessment of Disease Activity

Considering all the ways your arthritis affects you, rate how well you are doing on the following scale:

Very Well	O	O	O	O	O	O	O	O	O	O	O	O	O	O	O	O	O	O	O	O	O	Very Poor
	0	0.5	1.0	1.5	2.0	2.5	3.0	3.5	4.0	4.5	5.0	5.5	6.0	6.5	7.0	7.5	8.0	8.5	9.0	9.5	10	

Your Name______________________________ **Date of Birth** _________ **Today's Date** ___________

Provider Global Assessment of Disease Activity

Very Well	O	O	O	O	O	O	O	O	O	O	O	O	O	O	O	O	O	O	O	O	O	Very Poor
	0	0.5	1.0	1.5	2.0	2.5	3.0	3.5	4.0	4.5	5.0	5.5	6.0	6.5	7.0	7.5	8.0	8.5	9.0	9.5	10	

How to Score the CDAI

Variable	Range	Value
Tender joint score	(0-28)	
Swollen joint score	(0-28)	
Patient global score	(0-10)	
Provider global score	(0-10)	
**Add the above values to calculate the CDAI score**	**(0-76)**	

CDAI Score Interpretation	
0.0 – 2.8	Remission
2.9 – 10.0	Low Activity
10.1 – 22.0	Moderate Activity
22.1 – 76.0	High Activity

29. Did you experience morning stiffness within few days before/after onset of fever? **Y | N**
30. Did you experience pain in abdomen within few days before/after onset of fever? (Abdominal pain) **Y | N**
31. Did you experience nausea (Vomiting sensation) within few days before/after onset of fever? (Nausea) **Y | N**
32. Did you have vomiting within few days before/after onset of fever? (Vomiting) **Y | N**
33. Did you have diarrhea within few days before/after onset of fever? (3 or more loose/liquid stools/day)(Diarrhea) **Y | N**
34. Did you notice yellow discoloration of your skin or eye within few days before/after onset of fever? (Jaundice) **Y | N**
35. Did you experience any reduction in the level of alertness (somnolence to coma) within few days before/after onset of fever? (Altered sensorium) **Y | N**
36. Did you experience sudden uncontrollable muscle contraction/spasms otherwise known as seizures within few days before/after onset of fever? (Seizures) **Y | N**

    If Yes,

    36a. Type of seizure: ☐ Focal (Only a part of the body) ☐ Generalized (Whole Body)

    36b. How many times? ________
37. Did you experience partial or complete paralysis of any part of the body within few days before/after onset of fever? (Paralysis/Paresis) **Y | N**
38. Do you have any chronic/non communicable medical conditions?
    If yes, tick all applicable **Y | N**

38a. Hypertension (High Blood Pressure) : ☐
38b. Diabetes (sugar) : ☐
38c. Asthma/wheezing : ☐
38d. Liver Cirrhosis : ☐
38e. Chronic Renal failure : ☐
38f. Myocardial Infarction (heart attack) : ☐
38g. Stroke : ☐
38h. Cancer : ☐
38i. Others (Any other major illness diagnosed recently, Please mention__________________)

39. Is there anything that you wish to tell me which you think I have not asked you? **Y | N**
If yes brief:
____

____

EPIDEMIOLOGIC DATA

40. What is the source of drinking water in your home? (Tick all applicable)
☐ Dug well ☐ Hand pump ☐ Tube well ☐ Public well
☐ Public water tap ☐ Panchayath/ Municipality water supply
☐ Tanker water supply ☐ River, Pond, Stream ☐ Others: ____________________
40a. If it is Public tap/municipality water supply/ tanker water supply how often do you get water in a week?.................... Times per weeks
41. Do you store water in home? **Y | N**
If yes, 41a. where? (specify)____________
41b. How long? (specify)____________
42. Did you sleep under the bed net last night? **Y | N**
43. Do you use mosquito repellent in home? **Y | N**
44a. If yes, what do you use as a repellent?..........................
44. At any time in the past 12 months, has anyone come into your/the patient's dwelling to spray the interior walls against mosquitoes (or fleas)? **Y | N | UN**
44a. If yes, How many months ago was the dwelling last sprayed? ………….months
45. Did you have contact with anyone having/had similar illness in the month before you got sick? **Y | N**
If yes, Whom?....................
And When?.......................
And Where? ………………
46. Did you go for any travel in the last one month? **Y | N**
If Yes,
46a. From: _________________ To: _________________ Date: __________________
46b. From: _________________ To: _________________ Date: __________________
47. Is there anything that you wish to tell me which you think I have not asked you? **Y | N**
If Yes describe____

PHYSICAL EXAMINATION

48. Weight (Kg) : ___________
49. Height (Cm) : ___________
50. BP (S/D) : ______/_____ mm Hg
51. Pulse rate : ___________ per min
52. Respiratory rate: ___________ per min
53. Temperature : ___________ oC
54. Rash : **Y | N**

    If yes,

    54a. ☐ Macule ☐ Patch ☐ Papule ☐ Maculopapular ☐ Vesicle ☐ Bullae
    ☐ Pustule ☐ Erythema ☐ Petechiae ☐ Purpura ☐ Echymosis ☐ Others: ________
55. Joint swelling : **Y | N**
56. Joint Pain/ tenderness : **Y | N**
57. Cough : **Y | N**
58. Pallor : **Y | N**
59. Icterus : **Y | N**
60. Lymphadenopathy : **Y | N**
61. Edema : **Y | N**

    If yes, 61a. ☐ facial edema ☐ pedal edema ☐ others: ______________
62. Cyanosis: : **Y | N**
63. Conjunctival congestion/Red eye/ Sub conjunctival hemorrhage : **Y | N**
64. Ear discharge : **Y | N**
65. Oral ulcers : **Y | N**
66. Stomatitis : **Y | N**
67. Other Observations / Comments: ____

____

PHYSICAL EXAMINATION BY PHYSICIAN

68. Hepatomegaly Size: ________ cm
69. Splenomegaly Size: ________ cm
70. Altered Sensorium **Y | N**
    70a. If Yes, record Glasgow coma scale score

	Score	Scoring scheme	
Eye Response		Spontaneous eye opening	4 points
		Opens to verbal command, speech, or shout	3 points
		Opens to pain, not applied to face	2 points
		No eye opening	1 point
Verbal Response		Alert and oriented	5 points
		Confused conversation, but able to answer questions	4 points
		Inappropriate responses, jumbled phrases, but discernible words	3 points
		Incomprehensible speech	2 points
		No sounds	1 point
Motor Response		Obeys commands for movement fully	6 points
		Localizes to noxious stimuli	5 points
		Withdraws from noxious stimuli	4 points
		Abnormal flexion, decorticate posturing	3 points
		Extensor response, decerebrate posturing	2 points
		No response	1 point

71. Cranial nerve palsy If yes, 71a. Specify the nerve________________
72. Sensory deficit If yes, 72a. Specify: ___________________
73. Neck rigidity **Y | N**
74. Abnormal movements **Y | N**
    If yes, 74a. ☐ Chorea ☐ Athetosis ☐ Tremors ☐ Others: __________________

75. Other observations/ Comments:

____

____

____

LABORATORY INVESTIGATIONS

76	**Hematology Investigations**	Date:	Date:	Date:
77	Hb (g/dl)			
78	TLC			
79	DLC	N___ L___ M___ E___ B___	N___ L___ M___ E___ B___	N___ L___ M___ E___ B___
80	Platelet			
81	ESR (mm in 1st hour)			
82	Others			
83	**Biochemistry Investigations (Blood)**	Date:	Date:	Date:
84	Creatinine			
85	Total Protein			
86	Albumin			
87	Total Bilirubin			
88	Direct Bilirubin			
89	AST (SGOT)			
90	ALT (SGPT)			
91	Alkaline Phosphatase			
92	CPK-MB			
93	CRP			
94	RA Factor			
95	Calcium			
96	Phosphorous			
97	Others			
98	Hypoglycemia	**Y | N**	**Y | N**	**Y | N**
99	Hyponatremia	**Y | N**	**Y | N**	**Y | N**
100	Hypokalemia	**Y | N**	**Y | N**	**Y | N**
101	**Biochemistry Investigations (CSF)**	Date:	Date:	Date:
102	Glucose			
103	Protein			
104	Chloride			
105	CSF Cell Count	/mm^3	/mm^3	/mm^3
105a	If more than zero, CSF Cell type	N___ L___ E___	N___ L___ E___	N___ L___ E___

106.Name of Attending Physician : ________________ Contact no.: ______________

107.Name of interviewer : ________________ Signature: ___________________

108. Date of start of interview : ___/___/______

109. Date of completion of data collection in CRF : ___/___/______
